# Development of hepatocellular adenomas and carcinomas in mice with liver-specific G6Pase-α deficiency

**DOI:** 10.1242/dmm.014878

**Published:** 2014-09

**Authors:** Roberta Resaz, Cristina Vanni, Daniela Segalerba, Angela R. Sementa, Luca Mastracci, Federica Grillo, Daniele Murgia, Maria Carla Bosco, Janice Y. Chou, Ottavia Barbieri, Luigi Varesio, Alessandra Eva

**Affiliations:** 1 Laboratory of Molecular Biology, Istituto Gannina Gaslini, 16147 Genova, Italy; 2 Department of Pediatric Pathology, Istituto Gannina Gaslini, 16147 Genova, Italy; 3 Department of Surgical and Diagnostic Sciences (DISC), Anatomic Pathology Unit, University of Genova, 16132 Genova, Italy; 4 IRCCS AOU San Martino-IST, National Cancer Research Institute, 16132 Genova, Italy; 5 Section on Cellular Differentiation, Program on Developmental Endocrinology and Genetics, National Institute of Child Health and Human Development, National Institutes of Health, Bethesda, MD 20892-1830, USA; 6 Department of Experimental Medicine (DIMES), University of Genova, 16132 Genova, Italy

**Keywords:** Glycogen storage disease type 1a, Glucose-6-phosphatase-α, Animal model, Hepatomegaly, Hepatic steatosis, Hepatocellular adenoma, Hepatocellular carcinoma

## Abstract

Glycogen storage disease type 1a (GSD-1a) is caused by a deficiency in glucose-6-phosphatase-α (G6Pase-α), and is characterized by impaired glucose homeostasis and a high risk of developing hepatocellular adenomas (HCAs). A globally G6Pase-α-deficient (*G6pc*^−/−^) mouse model that shows pathological features similar to those of humans with GSD-1a has been developed. These mice show a very severe phenotype of disturbed glucose homeostasis and rarely live beyond weaning. We generated liver-specific G6Pase-α-deficient (LS‑*G6pc*^−/−^) mice as an alternative animal model for studying the long-term pathophysiology of the liver and the potential treatment strategies, such as cell therapy. LS‑*G6pc*^−/−^ mice were viable and exhibited normal glucose profiles in the fed state, but showed significantly lower blood glucose levels than their control littermates after 6 hours of fasting. LS‑*G6pc*^−/−^ mice developed hepatomegaly with glycogen accumulation and hepatic steatosis, and progressive hepatic degeneration. Ninety percent of the mice analyzed developed amyloidosis by 12 months of age. Finally, 25% of the mice sacrificed at age 10–20 months showed the presence of multiple HCAs and in one case late development of hepatocellular carcinoma (HCC). In conclusion, LS‑*G6pc*^−/−^ mice manifest hepatic symptoms similar to those of human GSD-1a and, therefore, represent a valid model to evaluate long-term liver pathogenesis of GSD-1a.

## INTRODUCTION

Glycogen storage disease type 1a (GSD-1a) is an autosomal recessive disorder caused by mutations in glucose-6-phosphatase-α (G6Pase-α; encoded by *G6PC*), an enzyme expressed primarily in gluconeogenic organs, such as the liver, kidney and intestine, where it catalyzes the hydrolysis of glucose-6-phosphate (G6P) to glucose in the terminal step of glycogenolysis and gluconeogenesis (Chou et al., 2010). GSD-1a causes glycogen accumulation in the liver and kidney, and prevents glucose release into the blood (Lei et al., 1996). The patients manifest hypoglycemia, growth retardation, hepatomegaly and nephromegaly with consequent compromised liver and kidney functions. The current treatment of GSD-1a is based on the control of symptomatic hypoglycemia by naso-gastric infusion of glucose and frequent oral administration of cornstarch (Greene et al., 1976; Chen et al., 1984). However, the underlying disease remains untreated, and patients still develop long-term complications such as hepatic adenomas, with risk of malignancy, and renal failure. Therefore, alternative treatment strategies are required.

Attempts to isolate the G6Pase-α protein in an active soluble form have been unsuccessful owing to its hydrophobicity, ruling out protein replacement therapy as a treatment option. Liver transplantation can modify the prognosis of the disease (Matern et al., 1999; Labrune, 2002), but it is a highly invasive procedure and carries complications inherent to long-term immunosuppressive therapy and the risk of rejection, providing only short-term solutions.

Alternative therapeutic approaches are represented by gene and cell therapy. A mouse model for GSD-1a, closely mimicking the human disorder, has been generated (Lei et al., 1996). Although long-term complications of GSD-1a cannot be studied because most of these animals die within 3 weeks after birth, this mouse model has been useful to develop somatic gene therapies. Studies showed that viral vectors can deliver the *G6PC* transgene into the liver of *G6pc*^−/−^ mice, improve their survival and correct metabolic abnormalities to various degrees (Zingone et al., 2000; Ghosh et al., 2006; Sun et al., 2002; Koeberl et al., 2006; Yiu et al., 2010; Lee et al., 2012). The most effective viral vector reported to date is a recombinant adeno-associated virus vector expressing G6Pase-α directed by the native *G6PC* promoter/enhancer that effectively extends the lifespan of mice, corrects the metabolic abnormalities and even prevents the development of hepatocellular adenoma (HCA) (Yiu et al., 2010; Lee et al., 2012).

We have analyzed the efficacy of gene delivery systems based on cell therapy to treat GSD-1a in the same *G6pc*^−/−^ mice. We showed that transplantation of CD11b+ cells, selected from bone marrow of congenic wild-type mice, into *G6pc*^−/−^ mice partially reconstitutes the diseased liver and prolongs lifespan (Resaz et al., 2011). The rather slow process involving CD11b+ cell reconstitution of the liver combined with the very severe phenotype displayed by *G6pc*^−/−^ mice, however, do not allow long-term survival. Therefore, long-term effects of CD11b+ cell transplantation, including potential consequences and/or side effects on the liver or other organs colonized by these cells, could not be studied.

**TRANSLATIONAL IMPACT**

**Clinical issue**

Glycogen storage disease type 1a (GSD-1a) is a rare hereditary syndrome with an overall incidence of approximately 1 in 100,000 individuals. GSD-1a is caused by an inactivating mutation in the gene *G6PC*, which encodes glucose-6-phosphatase-α (G6Pase-α), an enzyme that is expressed in gluconeogenic organs: the liver, kidney and intestine. Individuals with GSD-1a display hypoglycemia, hepatomegaly, nephromegaly, hyperlipidemia, hyperuricemia, lactic acidemia and growth retardation. The current treatment of GSD-1a is based on dietary therapy for the control of hypoglycemia, but long-term complications still develop, including hepatic adenomas (that might undergo malignant transformation) and renal disease, the two major causes of morbidity and mortality in human GSD-1a. A *G6Pc*-null mouse model for GSD-1a that closely mimics the human disorder has been generated previously. These animals have been very useful for the development of somatic gene therapies, but early death of these mice prevents them being used for long-term studies on disease-associated organ degeneration. Therefore, new animal models are needed to study the long-term liver pathogenesis of GSD-1a.

**Results**

In this study, the authors generated a mouse line carrying liver-specific deletion of G6Pase-α (LS-*G6pc*^−/−^). These mice display a milder pathological phenotype and improved survival in comparison with *G6Pc*-null mice. The authors characterized the progression of liver degeneration in LS-*G6pc*^−/−^ mice and demonstrated that these mice exhibit hepatic symptoms similar to those of human GSD-1a, including hepatomegaly, steatosis and glycogen accumulation. The LS-*G6pc*^−/−^ mice presented with hypercholesterolemia and hypertriglyceridemia until they reached the age of 2-4 months, and suffered from fasting hypoglycemia throughout their lives. Finally, 25% of LS-*G6pc*^−/−^ mice developed liver nodules by 10–20 months of age. These nodules were identified as adenomas and in one case as hepatocellular carcinoma.

**Implications and future directions**

Animal models are powerful tools to study hereditary diseases and guide the development of new therapeutic approaches. This work shows that LS-*G6pc*^−/−^ mice display liver abnormalities that closely resemble those of the human pathology. Additionally, because these animals can survive in the long term, they represent a valuable model to study the age-related disease progression, and potentially to discover biomarkers and new treatment interventions. In particular, this model might be useful to evaluate the long-term efficacy of gene and cellular therapies in preclinical studies, such as hematopoietic stem cell approaches to regenerate the liver of diseased animals.

To overcome this problem we have generated a mouse line (LS-*G6pc*^−/−^) carrying liver-specific deletion of G6Pase-α. We report here the characterization of these mice. LS-*G6pc*^−/−^ mice manifested a milder pathological situation in comparison with *G6pc*^−/−^ mice. Nevertheless, LS-*G6pc*^−/−^ mice exhibited hepatic symptoms similar to those of human GSD-1a and, therefore, represent a valid model to evaluate the correction of liver abnormalities by cell therapy and long-term consequences of CD11b+ cell engraftment. Moreover, these mice will be useful to study long-term liver pathogenesis of GSD-1a.

## RESULTS

### Generation of liver-specific *G6pc*^−/−^ mice

**Fig. 1.**
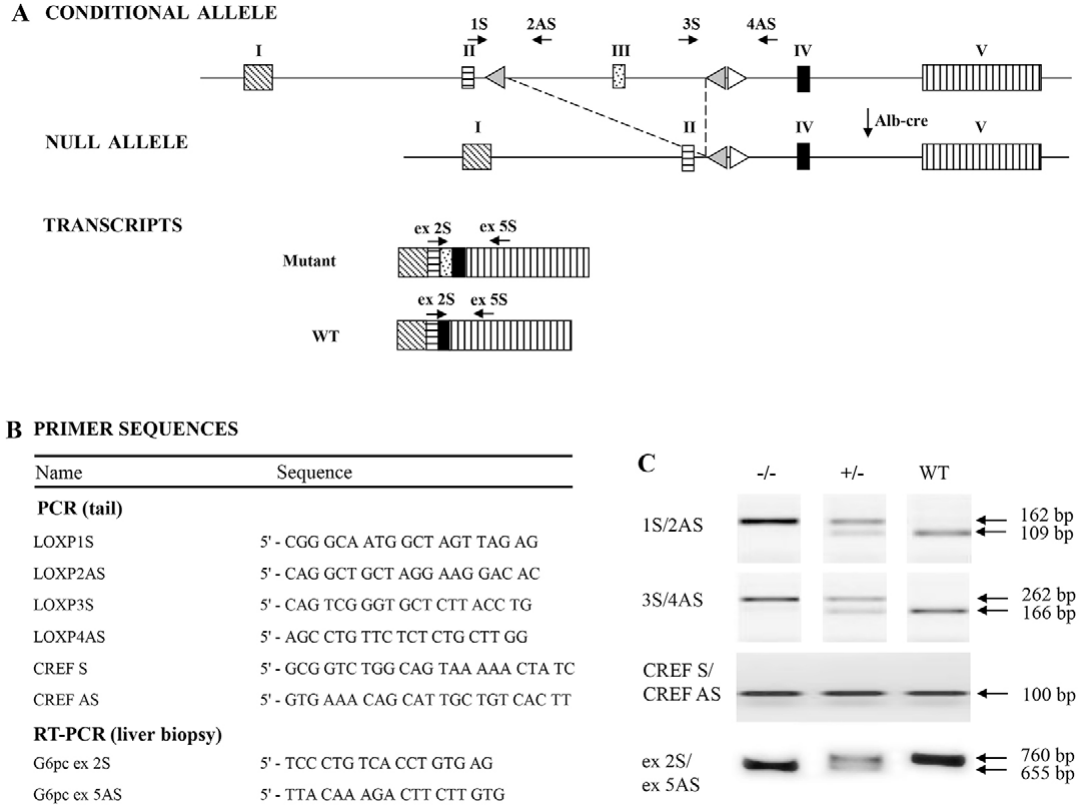
**Generation of liver-specific *G6pc*-null mice.** (A) Schematic representation of the *G6pc* conditional allele, the *G6pc* null allele, and the wild-type (WT) and mutant *G6pc* transcripts. Rectangles are exons; white triangles represent *loxP* sequences and gray triangles *frt* sequences. Arrows indicate the primers used to genotype the mice and verify exon 3 excision. (B) Name and sequence of the primers used for PCR on mouse tail DNA and RT-PCR on RNA extracted from liver biopsies. (C) PCR and RT-PCR analysis of WT and mutant alleles. The primer pair 1S/2AS is expected to amplify a fragment of 109 bp in the WT allele and a fragment of 162 bp in the mutant allele. The primer pair 3S/4AS is expected to amplify a fragment of 166 bp in the WT allele and a fragment of 262 bp in the mutant allele. The primer pair CREF S/CREF AS was used to detect a 100 bp Cre fragment. To verify liver-specific excision of exon 3, RT-PCR analysis was performed on RNA extracted from liver biopsy with the primer pair G6pc ex 2S/G6pc ex 5AS, expected to amplify a fragment of 760 bp in WT alleles and a fragment of 655 bp in the mutant allele. The images shown in the top two panels in C were selected from non-contiguous lanes of the same agarose gel and are representative of the results obtained.

To generate LS-*G6pc*^−/−^ mice, *G6pc^fx/fx^* mice, harboring a conditional null allele for *G6PC* (Peng et al., 2009), were crossed with transgenic B6.Cg-Tg(Alb-cre)21Mgn/J mice (Fig. 1A). B6.Cg-Tg(Alb-cre)21Mgn/J mice have been well characterized and shown to be nearly 100% efficient in achieving liver-specific recombination and thus in performing liver-specific gene knockouts (Postic et al., 1999). Mice were genotyped by PCR analysis of genomic DNA from mouse tails (Fig. 1). The liver-specific deletion of exon 3 was confirmed at the mRNA level by RT-PCR on RNA extracted from biopsies of liver tissues obtained from 2- to 3-day-old mice (Fig. 1C). A total of 125 mice of 2 to 3 days of age were analyzed in this way. In all cases genotypes obtained by PCR analysis of genomic DNA from mouse tails were confirmed by RT-PCR on RNA extracted from biopsies of liver tissues (supplementary material Fig. S1). We also determined that the efficiency of recombination in 2- to 3-day-old mice was more than 80% (supplementary material Fig. S2).

### Histology and histochemistry

**Fig. 2.**
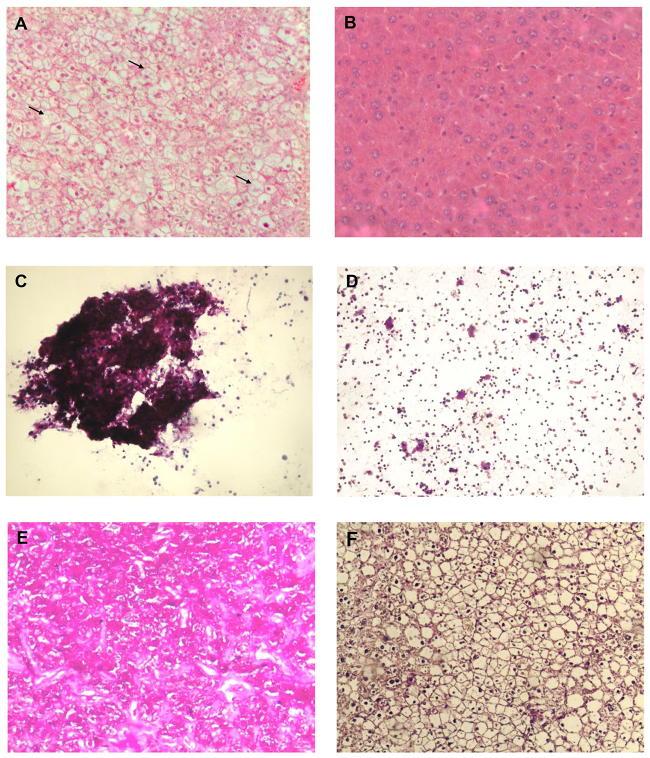
**Histological analysis of livers from LS-*G6pc*^−/−^ and control mice.** Paraffin-embedded H&E-stained liver sections from a 2-month-old LS-*G6pc*^−/−^ mouse (A) and control mouse (B). Arrows show macro- and micro-lipid vesicles in the enlarged hepatocytes. Liver fine-needle aspiration cytology sample stained with PAS from a 2-day-old LS-*G6pc*^−/−^ mouse (C) and a 2-day-old control mouse (D). Paraffin-embedded liver sections from 3-month-old LS-*G6pc*^−/−^ mice were stained with PAS (E) or PAS plus diastase (F). (A,B,E,F) 10× magnification; (C,D) 20× magnification.

**Fig. 3.**
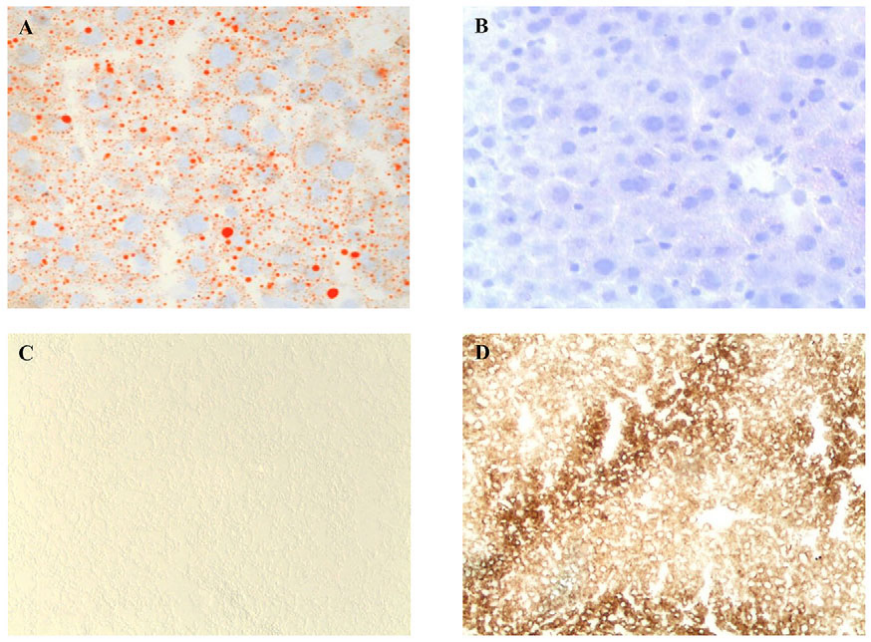
**Histochemical analyses of liver from LS-*G6pc*^−/−^ mice.** Oil red O staining of LS-*G6pc*^−/−^ mouse (A) or control mouse (B) liver cryostat sections at 2 months of age shows lipid accumulation in the hepatocytes of the LS-*G6pc*^−/−^ mouse. To evaluate G6Pase activity, liver cryostat sections were treated as described in the Materials and Methods. Colored lead sulfide was developed with ammonium sulfide. Representative results of liver sections from 3-month-old LS-*G6pc*^−/−^ (C) and control (D) mice are shown. (A,B) 20× magnification; (C,D) 10× magnification.

LS-*G6pc*^−/−^ mice were found to be viable and fertile and showed no growth retardation. However, these mice manifested a liver phenotype typical of GSD-1a, characterized by hepatomegaly. This clinical presentation in GSD-1a is primarily caused by excess of glycogen and lipid deposition. Fig. 2 shows the histological appearance of livers from 3-week-old LS-*G6pc*^−/−^ and control mice. LS-*G6pc*^−/−^ mice had marked glycogen deposition in hepatocytes (Fig. 2A). Steatosis in LS-*G6pc*^−/−^ mice was revealed by the presence of macro- and micro-lipid vesicles in the enlarged hepatocytes (Fig. 2A, arrows). Glycogen build-up in the liver of LS-*G6pc*^−/−^ mice was revealed by periodic acid–Schiff (PAS) staining of liver fine-needle aspiration cytology samples of 2- to 6-day-old LS-*G6pc*^−/−^ mice (Fig. 2C), and of liver sections of LS-*G6pc*^−/−^ adult mice (Fig. 2E). Diastase digestion removed the majority of the magenta staining from PAS-treated liver sections (Fig. 2F), confirming heavy accumulation of intracellular glycogen. Oil red O staining of liver sections showed large fat droplets within hepatocytes, confirming the marked lipid accumulation in comparison to control mice (Fig. 3A,B).

Hepatic G6pase activity was evaluated by histochemical detection in liver tissues of LS-*G6pc*^−/−^ mice. G6Pase activity was detectable in the livers of wild-type mice (Fig. 3D), in contrast to the livers of LS-*G6pc*^−/−^ mice for which staining was negative (Fig. 3C). These results indicate that functional G6Pase-α is undetectable in livers of LS-*G6pc*^−/−^.

**Fig. 4.**
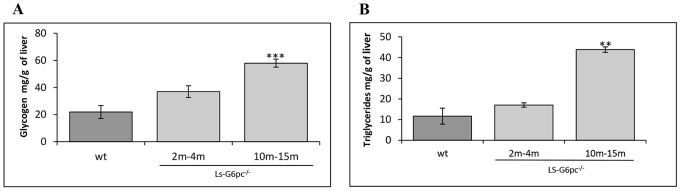
**Hepatic glycogen and lipid content of LS-*G6pc*^−/−^ mice.** Liver glycogen (A) and triglyceride (B) content of LS-*G6pc*^−/−^ mice were determined at the indicated months (m) of age. Each value represents the mean of the measurement of four or five mice. The values for control (wt) mice are the pooled data of animals aged 2 weeks to 3 months. Data are presented as mean ± s.d. Values that are significantly different from control mice (*P*<0.01** and ***P<0.005) are indicated.

The glycogen content in the livers of LS-*G6pc*^−/−^ mice was significantly higher than that of control mice, increasing with age to reach 60 mg/g of liver in 10- to 15-month-old mice (Fig. 4A). Similarly, accumulation of triglycerides in LS-*G6pc*^−/−^ livers significantly increased with age, in agreement with the marked Oil red O staining of liver sections (Fig. 4B).

### Metabolic characteristics of LS-*G6pc*^−/−^ mice

**Fig. 5.**
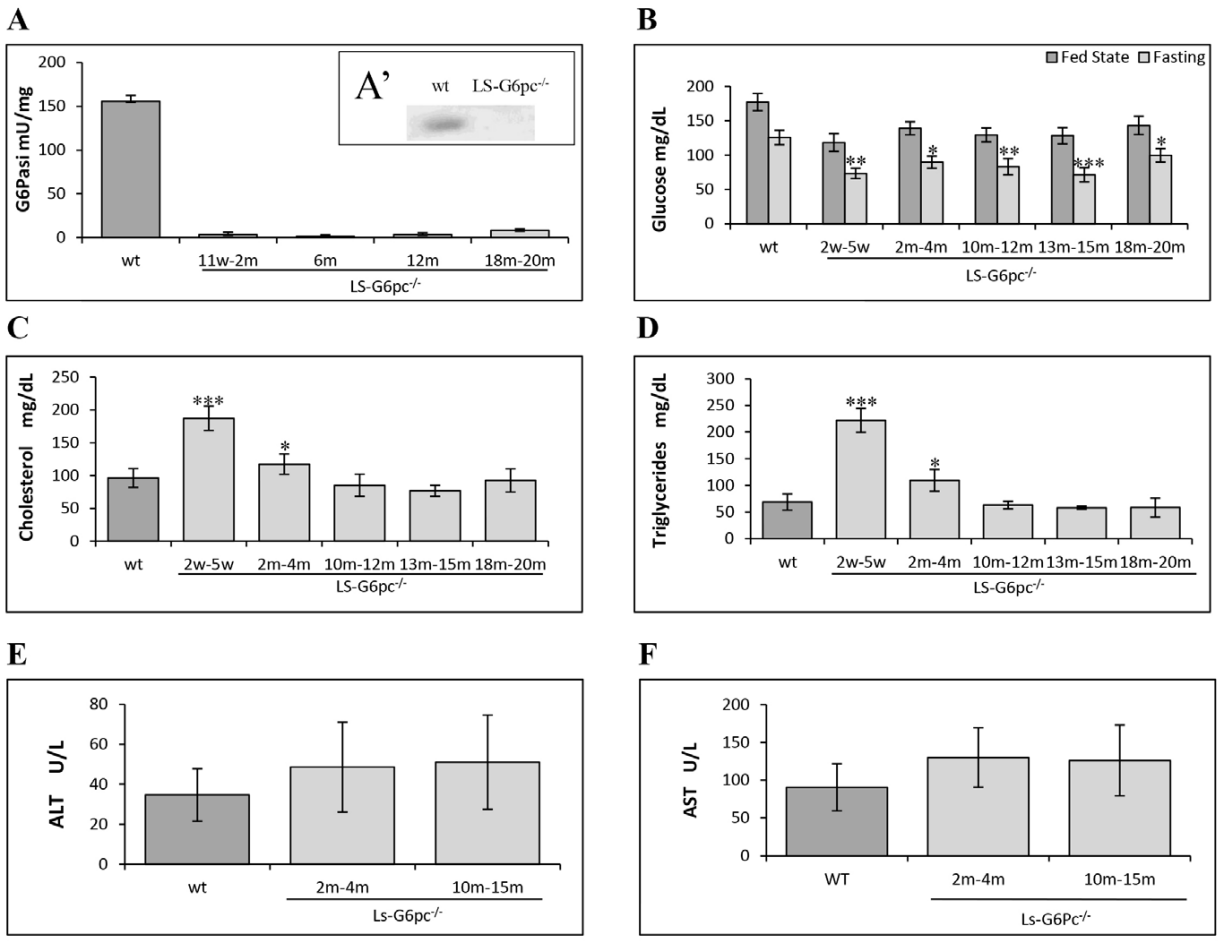
**Hepatic G6Pase activity and plasma metabolic parameters in LS-*G6pc*^−/−^ mice.** Three to five mice were analyzed for each group of LS-*G6pc*^−/−^ mice. G6P phosphohydrolase activity in the liver microsomes (A), and blood glucose (B), total cholesterol (C), triglyceride (D), ALT (E) and AST (F) levels of LS-*G6pc*^−/−^ mice were determined at the indicated ages in weeks (w) or months (m). Four age-matched wild-type mice were analyzed for each group. Because of the similarities of the respective metabolites in each group of control mice, results shown are pooled data of age 2 weeks-20 months. (A′) Liver lysates were subjected to western blotting and G6Pase-α expression was visualized with anti-G6Pase-α antibody. (B) Glucose levels were determined in the fed state (dark gray bars) or upon 6 hours of fasting (light gray bars). Data are presented as mean ± s.d. Values that are significantly different from control mice (*P*<0.05*, *P*<0.01** and ***P<0.005) are indicated.

We further analyzed microsomal G6P phosphohydrolase activity in the livers of LS-*G6pc*^−/−^ mice and compared it to that of age-matched wild-type littermates. As shown in Fig. 5A, G6P phosphohydrolase activity in the liver of LS-*G6pc*^−/−^ mice was undetectable. Moreover, western-blot analyses confirmed the absence of the G6PC protein in livers of LS-*G6pc*^−/−^ mice (Fig. 5A′).

Metabolic analysis revealed a fasting hypoglycemia typical of GSD-1a. The mean serum glucose level in LS-*G6pc*^−/−^ mice was significantly lower than that in control littermates after 6 hours of fasting (Fig. 5B). These mice, however, did not suffer from hypoglycemic seizure and therefore did not require glucose therapy, unlike *G6pc*^−/−^ mice (Lei et al., 1996). In fact, in the fed state, the mean blood glucose concentration in LS-*G6pc*^−/−^ mice was 25% lower than in control mice, but remained within the normal levels. Moreover, serum cholesterol and triglyceride mean levels were higher in 2- to 4-month-old LS-*G6pc*^−/−^ mice than in control littermates (Fig. 5C,D), but we observed improvement of serum cholesterol and triglyceride levels in mice older than 6 months (Fig. 5C,D).

Finally, serum levels of alanine aminotransferase (ALT) and aspartate aminotransferase (AST) were evaluated at 2–4 and 10–15 months of age (Fig. 5E,F). In general, transaminases were moderately to mildly increased in LS-*G6pc*^−/−^ mice as compared to healthy controls. Some mice, however, had normal levels of ALT and AST whereas some other mice showed high transaminase levels. The high standard deviation reflects this variability.

### Progressive liver damage with age

**Fig. 6.**
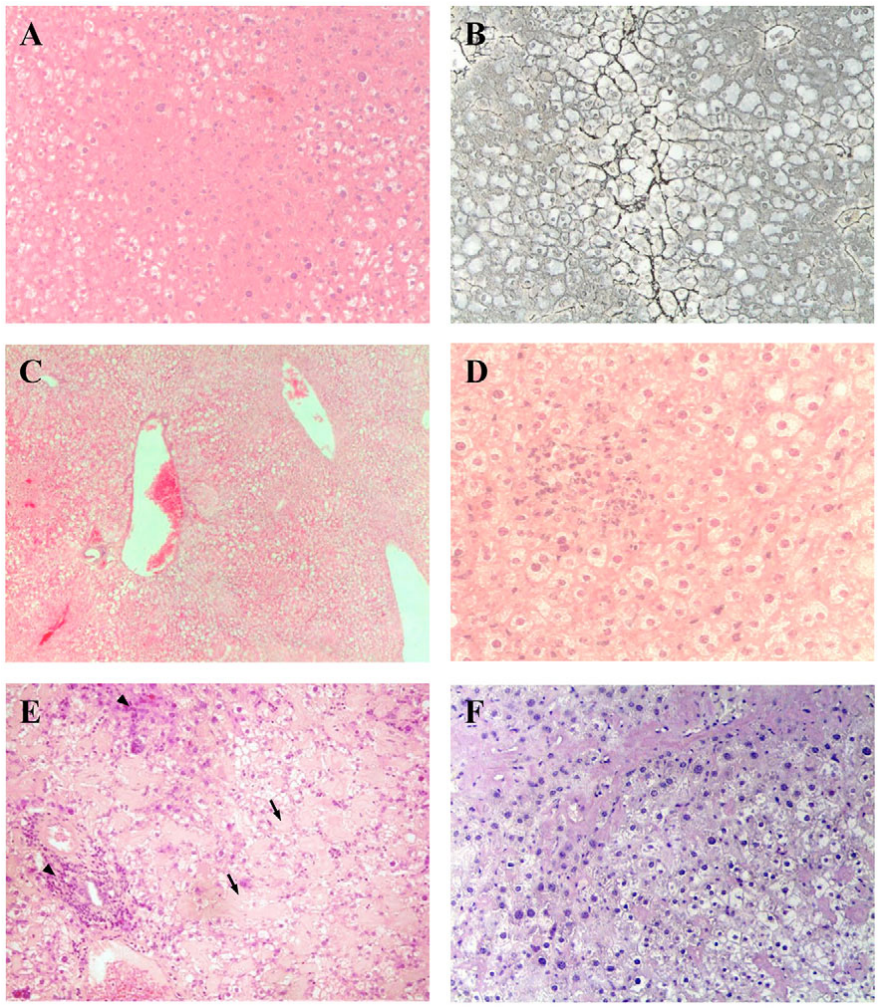
**Histological appearance of liver in adult mice.** (A) H&E and (B) reticulin staining shows foci of necrosis in a 2-month-old LS-*G6pc*^−/−^ mouse. (C) Liver section of a 4-month-old LS-*G6pc*^−/−^ mouse showing sinusoid and vein enlargement. (D) Liver section of an 8-month old LS-*G6pc*^−/−^ mouse with inflammatory infiltrates. (E) Liver section of a 15-month-old LS-*G6pc*^−/−^ mouse with inflammatory infiltrates (arrowheads) and sinusoidal amyloidosis (arrows) stained with H&E. (F) Extended amyloidosis is confirmed by Congo red staining. (A,B,E,F) 10× magnification; (C) 4× magnification; (D) 20× magnification.

The severity of liver damage increased as mice grew older. By 2 months of age areas of paucicellular necrosis with inflammatory cells started to appear (Fig. 6A,B), and initial damage of liver structure could be observed by sinusoid and vein enlargement (Fig. 6C).

In livers of older mice (aged 6 to 20 months), portal and periportal inflammatory infiltrates were detected (Fig. 6D). Moreover, marked amyloid deposition was observed in 90% of the livers of mice older than 10 months of age (Fig. 6E,F). Deposits were observed both in vessel walls and within sinusoids. The material stained positive with Congo red (Fig. 6F) and displayed green birefringence when viewed under polarized light (data not shown), confirming amyloid deposition.

**Fig. 7.**
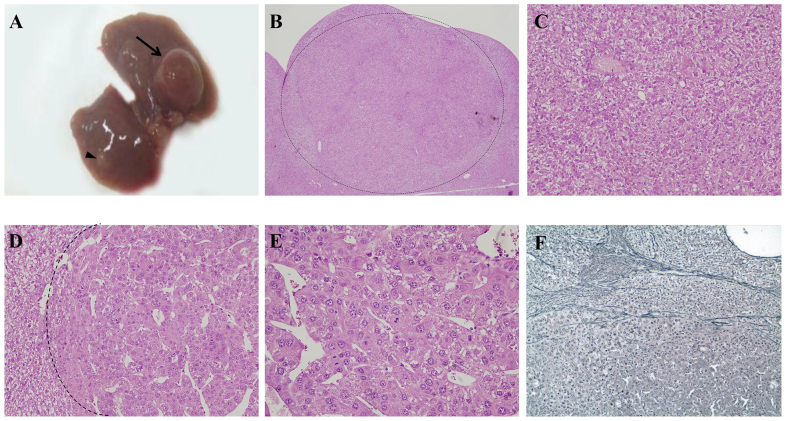
**Liver nodules in aging LS-*G6pc*^−/−^ mice.** (A) Liver resection of a 19-month-old LS-*G6pc*^−/−^ mouse with a 10 mm nodule (arrow). Smaller nodules are also present (arrowhead). (B) H&E-stained section from the liver of a 12-month-old LS-*G6pc*^−/−^ mouse showing a nodule histologically characterized as HCA (boundary shown as dotted line). (C) A higher magnification (H&E stained) of the same nodule showing normal hepatocytes, a solitary artery and no portal tracts, typical of HCA. (D) H&E-stained section of the liver of a 19-month-old LS-*G6pc*^−/−^ mouse showing a HCC within a HCA (boundary shown as dotted line). (E) Higher magnification of the HCC, showing thickened hepatocyte plates with trabecular and pseudoglandular architecture and evident cytological atypia with scattered mitoses. (F) HCC stained with reticulin stain, showing loss of reticulin framework typical of malignancy. Reticulin stain was preserved in the surrounding HCA. (B) 4× magnification; (C,D) 20× magnification; (E) 40× magnification; (F) 10× magnification.

HCA is a severe long-term complication of GSD-1a and develops in 70–80% of patients >25 years old (Chou et al., 2010). To determine whether LS-*G6pc*^−/−^ mice develop HCA, a group of 25 LS-*G6pc*^−/−^ mice and 25 wild-type age-matched littermates were analyzed. Two to 4 mice were sacrificed at various ages, ranging from 2 to 20 months, and their liver was analyzed for HCA or hepatocellular carcinoma (HCC) development. Five out of 19 LS-*G6pc*^−/−^ mice aged 10–20 months displayed detectable (2 mm) liver nodules (Table 1). One mouse developed a 5 mm nodule by 15 months of age. In one animal, aged 19 months, five small nodules (2–4 mm in diameter) (Fig. 7A, arrowhead) and a single large nodule of 10 mm (Fig. 7A, arrow) were detected. These nodules were characterized as HCA (Fig. 7B,C). They were unencapsulated and composed of hepatocytes with no atypia and no evidence of intralesional portal tracts. Within the largest HCA (10 mm), a 5 mm moderately differentiated HCC was detected (Fig. 7D,E). Malignant cells showed marked atypia and scattered mitoses, and a trabecular and pseudoglandular architecture was present. Whereas reticulin staining was preserved in the HCA, this focus of malignant transformation was further characterized by loss of reticulin framework (Fig. 7F). An interesting finding is that the focus of HCC showed no glycogen accumulation compared with the surrounding HCA.

**Table 1.**
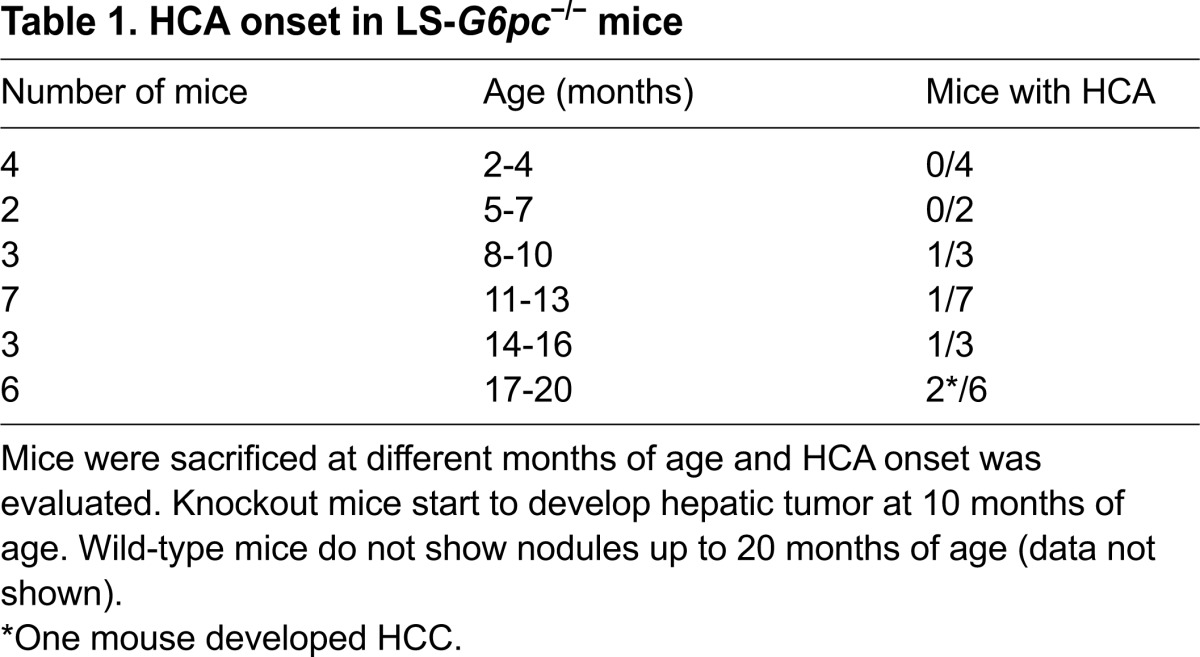
HCA onset in LS-*G6pc*^−/−^ mice

## DISCUSSION

We describe here the generation and characterization of a new mouse model for GSD-1a, in which *G6pc* deletion occurs only in the liver. These mice manifested most of the hepatic symptoms of the human pathology, including hepatomegaly, steatosis and glycogen accumulation. Blood glucose profiles of LS-*G6pc*^−/−^ mice in the fed state were within normal values, but consistently lower than their control littermates. Whereas control mice can tolerate 6 hours of fasting, the LS-*G6pc*^−/−^ mice suffered from the fasting hypoglycemia throughout their lives.

LS-*G6pc*^−/−^ mice also showed high plasma metabolic levels for cholesterol and triglycerides until they reached the age of 2-4 months. These values progressively decreased with time and reached normal levels in 6- to 8-month-old mice. It is reasonable to assume that some compensatory mechanisms occur as the mice get older, perhaps because of the normal function of the other two gluconeogenic organs, the kidney and the intestine.

It was previously reported that both *G6pc*^−/−^ mice and GSD-1a patients exhibit increased peripheral neutrophil counts and elevated serum cytokine levels (Kim et al., 2007), and that hepatic injury in *G6pc*^−/−^ mice is evidenced by necrosis and a markedly elevated level of infiltrating neutrophils (Kim et al., 2008). Similarly, livers of LS-*G6pc*^−/−^ mice showed marked steatosis, inflammation and necrosis. The average values of ALT and AST were above normal, as compared with healthy controls, even though we found variability of transaminase values among the mice analyzed, in agreement with what is reported for GSD-1a patients (Moraru et al., 2007; Wakid et al., 1987). This variability probably reflects the degrees of steatosis and glycogen accumulation, and the extent of areas of necrosis and inflammation, in the liver of LS-*G6pc*^−/−^ mice.

By 10 months of age, LS-*G6pc*^−/−^ mice developed liver nodules that were diagnosed as HCA and, in one case, as HCC within a HCA. Thus, even if the metabolic parameters tend to normalize as the mice age, hepatic degeneration worsened and the progressive degeneration of the liver led to premalignant and malignant transformation. Therefore, LS-*G6pc*^−/−^ mice manifested most of the liver damages of GSD-1a and developed hepatic long-term complications such as those affecting individuals with GSD-1a.

Finally, LS-*G6pc*^−/−^ mice developed marked vascular and sinusoidal amyloidosis. To our knowledge, amyloidosis has been reported only once in GSD-1b patients (Poe and Snover, 1988) and never in GSD-1a patients or in the other GSD-1a animal models. However, the presence of amyloidosis in the livers of LS-*G6pc*^−/−^ mice remains and further studies will be necessary to determine the reasons for the occurrence of this pathology.

GSD-1a is a rare disorder caused by a deficiency in G6Pase-α. Metabolic disruption in individuals with GSD-1a results in severe hypoglycemia and it is lethal in childhood if untreated. Nowadays, the disease can be efficiently managed with a rigorous diet and adjuvant drug therapy, prolonging long-term survival. Unfortunately, the chronic dysmetabolism results, with a patient’s aging, in a progressive worsening of the clinical parameters, increasing the need for medical treatment. Liver transplantation can modify the disease prognosis (Kirschner et al., 1991; Matern et al., 1999), but this procedure has severe limitations.

*G6pc*^−/−^ mouse (Lei et al., 1996) and dog (Kishnani et al., 2001) models, both closely mimicking the human disorder, have been generated, with the intent to delineate the disease more closely and to evaluate new therapeutic strategies. Although these animals have been used to develop somatic gene therapies that show promise as being efficacious treatments for GSD-1a (Chou et al., 2010; Resaz et al., 2011), they are unsuitable for long-term studies on disease-associated organ degeneration owing to their early death if untreated. Therefore, there is the need for alternative animal models for GSD-1a for studying both long-term liver degeneration and long-term effects of new therapeutic approaches.

To this end, Mutel et al. (Mutel et al., 2011) recently generated an inducible liver-specific *G6pc*-null mouse that exhibits a normal survival rate compared with *G6pc*^−/−^ mice. The deletion of *G6pc* was induced in adult mice, causing liver degeneration and development of HCAs in 100% of the mice by 18 months after gene deletion. These results show that deletion of the gene in a fully developed mouse still causes major defects in the liver, including steatosis and glycogen accumulation. These defects are similar to those we observed by knocking out the gene in the fetal liver or to those observed after inactivating the gene in the whole mouse embryo (Lei et al., 1996). The dysmetabolism caused by knocking out *G6pc* in the adult mouse (Mutel et al., 2011) seems to trigger uncontrolled adenoma formation in the liver of every mouse. In contrast, LS-*G6pc*^−/−^ mice, aged 10–19 months, deficient of G6Pase-α in the liver from birth, developed adenomas with a frequency of about 25%. One explanation is that the lack of the gene at birth can be more readily compensated by other biochemical pathways or by the function of the intestine and kidney, which are the other two gluconeogenic organs, thereby reducing the frequency of adenoma formation. Moreover, the technique used by Mutel et al. (Mutel et al., 2011) to detect HCA, i.e. magnetic resonance imaging, allowed the group to monitor the animals throughout their lives and therefore to evaluate the development of very small nodules as early as 9 months. The use of this powerful imaging technique might account, in part, for these differences. On the other hand, LS-*G6pc*^−/−^ mice displayed hepatic damage at birth and, therefore, should represent more closely the conditions of GSD-1a patients. In this respect, we observed that HCAs can develop into HCC, a pathological condition affecting 10% of GSD-1a patients (Rake et al., 2002; Lee, 2002; Franco et al., 2005). Therefore, our animal model for GSD-1a is unique because: (i) it is the only model with a constitutive liver deletion of *G6pc* that allows the high mortality of the knockout model to be overcome (Lei et al., 1996) and permits the study of the GSD-1a liver disease without delaying the onset of the deletion; and (ii) it is the only one so far described that mimics all the steps of the disease progression, including the development of HCC, providing experimentally a link between G6Pase-α deficiency and neoplastic liver progression.

Aging GSD-1a patients exhibit marked variability in the progression of the severity of symptoms and complications, including the link between the onset of HCA and HCC and the metabolic control of the disease, the presence of systemic indices of inflammation, and the renal pathogenesis and its correlation with the metabolic imbalance. The underlying pathological pathways that develop with age are poorly understood, and there are no biomarkers associated with the variability of the progressive tissue alteration.

We have developed a new and clinically relevant murine model that has the potential to provide insights into the progressive age-dependent liver degeneration in GSD-1a, because these mice display variability in the severity of the complications and the long-term consequences of the hepatic degeneration, similar to what is observed in individuals with GSD-1a. Moreover, this mouse model will also be useful for preclinical, long-term studies of gene- and cellular-therapy efficacy.

## MATERIALS AND METHODS

### Generation of a mouse line carrying liver-specific deletion of the gene encoding G6Pase-α

Generation of mice with a conditional null allele for *G6pc* (*G6pc* *^fx/fx^*) was previously reported (Peng et al., 2009). The LS-*G6pc*^−/−^ mice were generated by crossing the *G6pc* *^fx/fx^* mice with B6.Cg-Tg(Alb-cre)21Mgn/J transgenic mice (Jackson Laboratories), expressing the Cre recombinase under the control of the mouse albumin enhancer/promoter for liver-specific expression (Fig. 1A). Mouse genotypes were determined by PCR on mouse tail genomic DNAs with the two primer pairs 1S/2AS and 3S/4AS (Fig. 1B,C). The presence of Cre was confirmed by PCR on mouse tail DNAs with the primer pair CREF S and CREF AS (Fig. 1B,C). RT-PCR analysis for deletion of exon 3 was performed with RNA, extracted from liver biopsy by fine-needle aspiration, with the primer pair G6pc ex 2S/G6pc ex 5 AS (Fig. 1B,C and supplementary material Fig. S1). To estimate the percentage of undeleted exon 3, cDNAs derived from 1 μg of RNAs extracted from liver biopsies of 2- to 3-day-old mice containing the Alb-Cre transgene and homozygous for the mutant allele were mixed with increasing amounts of cDNAs derived from 1 μg of RNA extracted from liver biopsies of 2- to 3-day-old wild-type mice and subjected to PCR analysis with the primer pair G6pc ex 2S/G6pc ex 5 AS (supplementary material Fig. S2).

All animal studies were reviewed and approved by the Ethical Committee for Animal Experimentation (CSEA) as Animal Use project n. 291 communicated to the Italian Ministry of Health having regard to the article of the D.lgs 116/92, and carried out at the animal facility of the National Institute for Cancer Research (Genova, Italy).

### Histology and histochemistry

Fine-needle aspiration was performed on 2- to 6-day-old mice. Tissues were fixed in 10% neutral buffered formalin, embedded in paraffin and cut into 3–5 μm sections. Tissue sections were then deparaffinized, rehydrated and stained with hematoxylin/eosin (H&E), periodic acid-Schiff (PAS), PAS diastase, reticulin stain and Congo red. Staining kits were purchased from Ventana Medical Systems, Tucson, AZ, USA, and utilized according to the manufacturer.

### Phenotype analysis

For G6Pase histochemical assay, cryostat sections were incubated for 10 minutes at room temperature in 40 mM Tris-maleate buffer, pH 6.5, 300 mM sucrose, 10 mM glucose-6-P, 3.6 mM Pb(NP_3_)_2_. Sections were rinsed in 300 mM aqueous sucrose solution for 1 minute, lead sulfide was developed in a 1:100 diluted aqueous solution of 27% ammonium sulfide solution for 1 minute and then rinsed in 300 mM aqueous sucrose solution for 1 minute. Microsomes were prepared from frozen portions of livers collected from control or LS-*G6pc*^−/−^ mice, as previously described (Lei et al., 1996; Hiraiwa et al., 1999). For the phosphohydrolase assay, appropriate amounts of microsomal proteins were incubated at 30°C for 10 minutes in a reaction mixture (100 μl) containing 50 mM sodium cacodylate buffer, pH 6.5, 10 mM G6P, and 2 mM EDTA. Sample absorbance was determined at 820 nm and was related to the amount of phosphate released using a standard curve constructed with a stock of inorganic phosphate solution. For Oil red O staining, 6-μm cryostat sections were fixed in 10% formalin on ice and incubated first in 100% propylene glycol for 5 minutes, than in Oil red solution for 10 minutes, and finally in 85% propylene glycol for 5 minutes.

Mouse blood samples were collected by tail tip bleeding under anesthesia. Alternatively, mice were anesthetized and blood was drawn from the left ventricle with a syringe. Glucose levels were analyzed with Accu-chek Aviva (Roche Diagnostics, Indianapolis, IN, USA). Serum triglycerides and cholesterol levels were analyzed using kits purchased from IDLabs Biotechnologies London, ON, Canada. Serum alanine transaminase (ALT) and aspartate transaminase (AST) levels were determined using IDTox ALT Enzyme Assay Kit and IDTox AST Enzyme Assay Kit (IDLabs Biotechnologies London, ON, Canada). Liver glycogen was measured using EnzyChrom Glycogen Assay kit (Bioassay Systems, Hayward, CA, USA) and triglyceride content was determined on frozen tissue as described by Yamaguchi and Nakagawa (Yamaguchi and Nakagawa, 2007) using IDTox Triglyceride Enzyme assay kit (IDLabs Biotechnologies London, ON, Canada) according to the manufacturers’ instructions.

### Western blot analysis

Appropriate amounts of microsomal proteins (100 μg), obtained from control and LS-*G6pc*^−/−^ livers, were subjected to 10% SDS-PAGE electrophoresis, transferred to PVDF (Millipore, Billerica, MA, USA) and probed with a polyclonal anti-G6Pase-α antibody (Santa Cruz Biotechnology, Dallas, TX, USA). Immunocomplexes were visualized by West Dura extended chemiluminescent detection (Thermo Scientific, Walthman, MA, USA) using a HRP-conjugated secondary antibody (Pierce, Rockford, IL, USA,).

### Statistical analyses

Results are reported as means ± s.d. The unpaired *t*-test was performed using the GraphPad Prism Program, version 3.2 (GraphPad Software, San Diego, CA, USA). Values were considered statistically significant at *P*<0.05.

## Supplementary Material

Supplementary Material
