# Supplementary material for: Development of hepatocellular adenomas and carcinomas in mice with liver-specific G6Pase-α deficiency
Source: Dis Model Mech. 2014 Sep;7(9):1083–91. doi: 10.1242/dmm.014878 (PMC4142728; doi:10.1242/dmm.014878)
Supplement: Supplementary Material [file supp_7_9_1083__index.html]

Development of hepatocellular adenomas and carcinomas in mice with liver-specific G6Pase-α deficiency — Supplementary Material 

# Development of hepatocellular adenomas and carcinomas in mice with liver-specific G6Pase-α deficiency

## DMM014878 Supplementary Material

**Files in this Data Supplement:**

- **Supplementary Material**
