# Supplementary material for: Development of hepatocellular adenomas and carcinomas in mice with liver-specific G6Pase-α deficiency
Source: Dis Model Mech. 2014 Sep;7(9):1083–91. doi: 10.1242/dmm.014878 (PMC4142728; doi:10.1242/dmm.014878)
Supplement: Supplementary Material [file supp_7.9.1083_DMM014878.pdf]

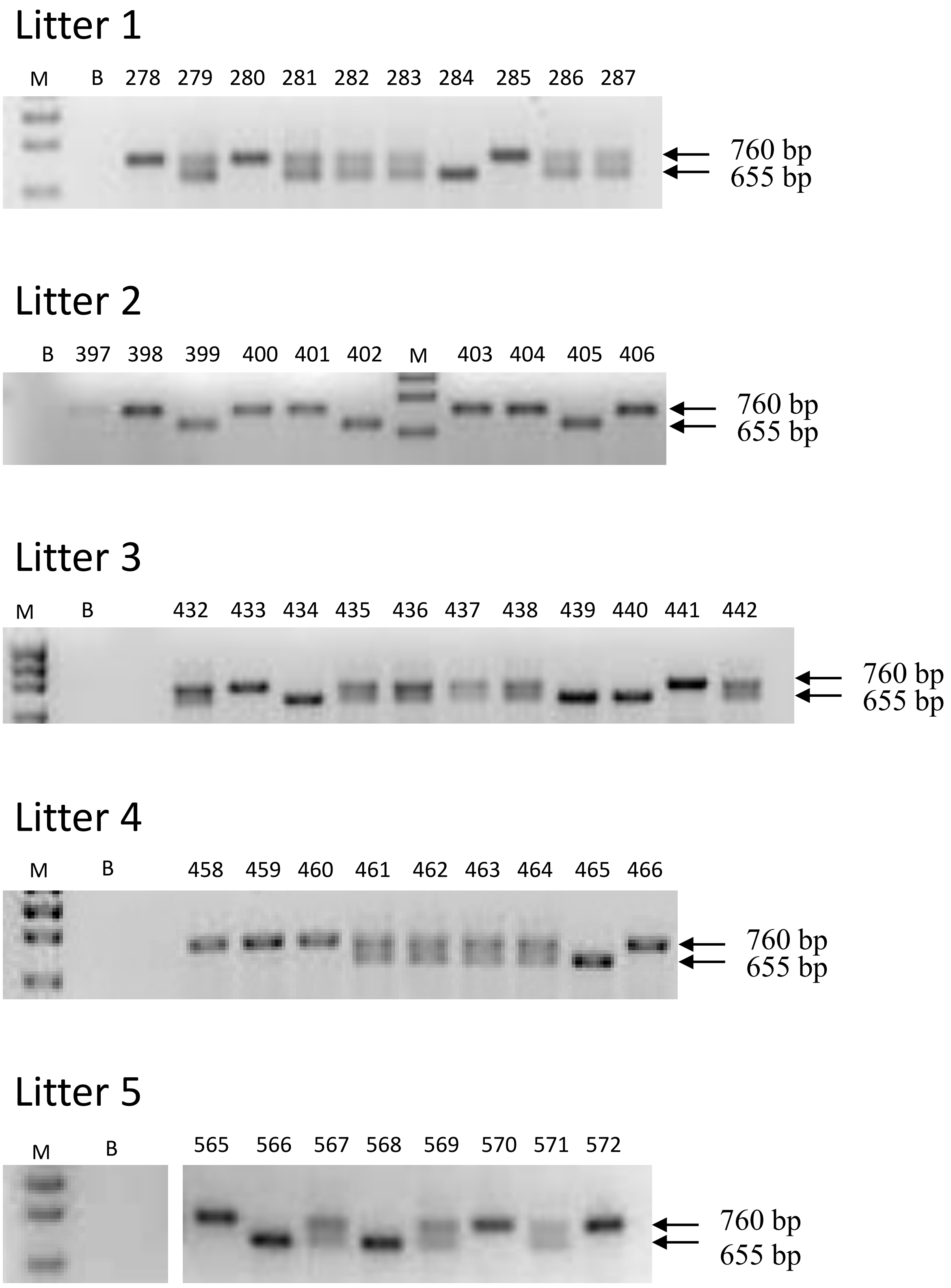

Figure S1. Liver-specific excision of exon 3. RT-PCR analysis was performed on RNA extracted from liver biopsy of 2-3 day old mice with the primer pair G6pc ex 2S/G6pc ex 5AS, expected to amplify a fragment of 760 bp in WT alleles and a fragment of 655 bp in the mutant allele. Results obtained with 5 different litters are shown. Mice identification numbers are shown. B, blank; M, DNA markers.

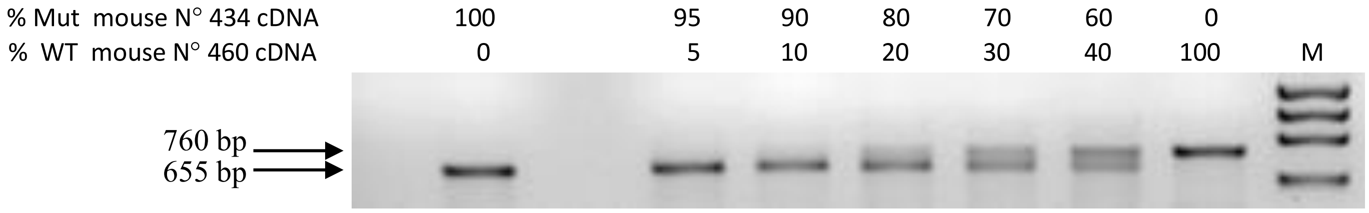

Figure S2. Evaluation of exon 3 excision by Cre. cDNA derived from 1 mg of RNAs extracted from liver biopsy of 2 day old mouse containing the Alb-Cre transgene and homozygous for the mutant allele (Mut) (mouse identification number 434) were mixed with the indicated increasing amounts of cDNA derived from 1 mg of RNA extracted from liver biopsy of 2 day old WT mouse (mouse identification number 460) and subjected to PCR analysis with the primer pair G6pc ex 2S/G6pc ex 5 AS, expected to amplify a fragment of 760 bp in WT alleles and a fragment of 655 bp in the mutant allele. M, DNA markers.
